# Cognitive Enhancement *via* Neuromodulation and Video Games: Synergistic Effects?

**DOI:** 10.3389/fnhum.2020.00235

**Published:** 2020-06-19

**Authors:** Marc Palaus, Raquel Viejo-Sobera, Diego Redolar-Ripoll, Elena M. Marrón

**Affiliations:** Cognitive NeuroLab, Faculty of Health Sciences, Universitat Oberta de Catalunya (UOC), Barcelona, Spain

**Keywords:** cognitive enhancement, dorsolateral prefrontal cortex, executive functions, iTBS, theta-burst stimulation, transcranial magnetic stimulation, video games, working memory

## Abstract

Transcranial magnetic stimulation (TMS) is a non-invasive brain stimulation technique able to modulate cortical excitability. This modulation may influence areas and networks responsible for specific cognitive processes, and the repetition of the induced temporary changes can produce long-lasting effects. TMS effectiveness may be enhanced when used in conjunction with cognitive training focused on specific cognitive functions. Playing video games can be an optimal cognitive training since it involves different cognitive components and high levels of engagement and motivation. The goal of this study is to assess the synergistic effects of TMS and video game training to enhance cognition, specifically, working memory and executive functions. We conducted a randomized 2 × 3 repeated measures (stimulation × time) study, randomly assigning 27 healthy volunteers to an active intermittent theta-burst stimulation or a sham stimulation group. Participants were assessed using a comprehensive neuropsychological battery before, immediately after, and 15 days after finishing the video game+TMS training. The training consisted of 10 sessions where participants played a 3D platform video game for 1.5 h. After each gaming session, TMS was applied over the right dorsolateral prefrontal cortex (DLPFC). All participants improved their video gaming performance, but we did not find a synergistic effect of stimulation and video game training. Neither had we found cognitive improvements related to the stimulation. We explored possible confounding variables such as age, gender, and early video gaming experience through linear regression. The early video gaming experience was related to improvements in working memory and inhibitory control. This result, although exploratory, highlights the influence of individual variables and previous experiences on brain plasticity.

## Introduction

Non-invasive brain stimulation techniques have become a step forward in cognitive neuroscience due to their ability to establish causal links between cognition and its neural substrate. Among these techniques, transcranial magnetic stimulation (TMS) allows modulation of cortical excitability in highly specific target regions, inducing changes in the associated cognitive functions and even enhancing them (e.g., Luber and Lisanby, 2014).

Nevertheless, the specific parameters through which TMS affects cognition are not entirely clear. TMS effectiveness seems to be partially task-dependent (Koch and Rothwell, 2009; Johnson et al., 2012; Duecker et al., 2013; Matsugi et al., 2014) and most effective when used together with cognitive training (Bentwich et al., 2011; Schilberg et al., 2012; Hopfner et al., 2015; Rabey and Dobronevsky, 2016; Lee et al., 2017; Nguyen et al., 2017). But both, the stimulation and the training must have certain characteristics to achieve the desired near-transfer and far-transfer effects (i.e., translation of benefits to similar or different cognitive domains, respectively).

Regarding the stimulation, its influence can be maximized when it is delivered after skill training. This allows us to take advantage of the state-dependency effects in specific neural populations (Romei et al., 2016), and TMS can interact with their current, imbalanced state (Silvanto et al., 2018). Examples in animal studies, using hypothalamic intracranial self-stimulation have shown that, administering the stimulation immediately after a skill training produce higher retention rates for that skill than administering the stimulation before the training (Redolar-Ripoll et al., 2002).

On the other hand, transfer effects of the training are maximized when different cognitive skills are integrated (Taatgen, 2013), high levels of engagement and motivation are maintained (Maraver et al., 2016), and there is sufficient exposure to the task (Zhao et al., 2020). In recent decades, video games have received a great deal of attention as cognitive training tools, due to some features that make them suitable for cognitive enhancement: they are widely available, integrate several cognitive processes at once, allow adjustment of variable difficulty, and are highly motivating and engaging. Furthermore, they are often used for long enough over a person’s lifetime to have a real impact on cognition. There is a considerable body of literature dedicated to the effects of video gaming on the brain (for a systematic review see Palaus et al., 2017), and the implications of using a particular video game genres are well understood (Dobrowolski et al., 2015).

Given the ability of TMS to induce plastic changes in the brain, and the particular suitability of video games to train cognitive functions, we expect that their combination would produce synergistic effects on cognitive enhancement, but the literature documenting combined use of TMS and video games is still scarce (e.g., Anguera et al., 2013). In particular, we expect to enhance cognition (i.e., processing speed, visuospatial skills, attention, working memory, executive functions, and general intelligence) in a healthy sample by playing a 3D platform video game during 10 sessions and applying TMS over the dorsolateral prefrontal cortex (DLPFC) immediately after playing. We hypothesized that post video game TMS would enhance the positive effects of video game training over cognition.

## Materials and Methods

### Participants

While 32 participants were recruited, five did not complete all the phases of the study (two due to discomfort during the stimulation, two due to incompatibility of schedules, and 1 for undisclosed personal reasons). The final sample was therefore composed of 27 healthy adults (14 women and 13 men) aged 18–40 years (29.44 ± 6.28). Participants were excluded if they had neurological or psychiatric disorders, including depression, measured through Beck’s Depression Inventory (BDI-II, Beck et al., 1996), were abusers of drugs or alcohol, did not meet safety criteria for both magnetic resonance imaging (MRI) and TMS (Rossi et al., 2009), and played video games (of any kind) for more than 3 h/week at the time of the study. Those who had previously played the video game used in this study (*Super Mario 64*) or any of its sequels, regardless of the experience level, were also excluded.

The study was approved by the Universitat Oberta de Catalunya (UOC) Ethics Committee. All participants gave written informed consent to participate in the study following the Declaration of Helsinki and received monetary compensation amounting to 80 euros.

### Design

A randomized 2 × 3 repeated measures (stimulation × time) study was conducted. Participants were randomly allocated to one of two stimulation groups: Active or Sham and evaluated before, immediately after the training period, and 15 days after finishing the training. When comparing the outcome measures of the two experimental groups, differences after training were only found in one of the variables (see “Results” section). Therefore, we decided to further explore the data to identify possible confounding variables that might have influenced the results.

Logistic regression was implemented to identify potential predictors of cognitive changes after the TMS+VG training period (see “Data Analysis” and “Results” section). Based on previous literature on video gaming and its effects on brain structure and function (see Palaus et al., 2017), we considered age, gender, and early video gaming experience as possible predictor variables. In line with previous studies (Hartanto et al., 2016; Palaus et al., 2017), early video game experience was defined as having played regularly before adolescence (14 years old or younger) for at least 1 year and more than 3 h/week (information collected through an *ad hoc* guided interview). The results of the logistic regression showed that early video gaming experience (in any kind of video game genre) predicted some of the performance changes observed in executive function tasks (see “Results” section). We, therefore, used this variable to classify the sample into four subgroups (see Table 1) according to stimulation modality (Active vs. Sham) and video game experience (Exp vs. NoExp).

**Table 1:** Characteristics of the groups (size, age ± standard deviation, and gender).

	Active	Sham	TOTAL
Experienced gamer	*n* = 6 (1 female) Age: 28.11 ± 7.23	*n* = 6 (2 female) Age: 28.33 ± 7.32	*n* = 12 (3 female) Age: 28.17 ± 7.30
Non-experienced gamer	*n* = 8 (6 female) Age: 32.50 ± 3.70	*n* = 7 (5 female) Age: 29.72 ± 7.00	*n* = 15 (11 female) Age: 30.47 ± 5.38
TOTAL	*n* = 14 (7 female) Age: 29.86 ± 5.26	*n* = 13 (7 female) Age: 29.00 ± 7.43	*n* = 27 (14 female) Age: 29.44 ± 6.28

### Procedure

**Figure 1:**
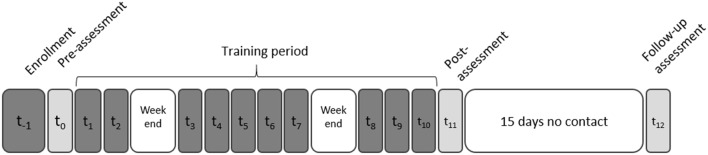
Experimental timeline: neuropsychological assessments (light gray), video game training and transcranial magnetic stimulation (TMS) sessions (dark gray), and non-contact days (white). A structural magnetic resonance imaging (MRI) was obtained for each participant during the enrollment phase to: (1) discard the possibility of brain anomalies that could affect or prevent participation; and (2) locate the stimulation target and navigate the TMS coil position for each participant (see “Transcranial Magnetic Stimulation” section).

The participants were enrolled in the study for 1 month, during which they participated in 10 TMS+VG sessions. They were assessed at three time points: before training started (Pre) after training ended (Post1), and 15 days after the end of the training (Post2; see Figure 1 and Table 2). Assessments and training procedures are described in what follows.

**Table 2:** Experimental schedule and phases following SPIRIT recommendations (Chan et al., 2013).

	Study period						
	Enrollment	Pre-assessment	TMS+VG training period			Post-assessment Time Point	
Time point	*t*_(−1)_	*t*_(0)_	*t*_(1)_	*t*_(2)_-*t*_(9)_	*t*_(10)_	*t*_(11)_ (post)	*t*_(12)_ (15-day follow-up)
**Recruitment and allocation**						
Eligibility screening	X						
Magnetic resonance imaging	X						
Informed consent		X					
Sociodemographic and video gaming data collection		X					
Group allocation		X					
**Training**						
Video gaming (1.5 h)							
TMS							
**Assessment**						
Active motor threshold			X				
TMS screening			X	X	X		
Video game survey			X	X	X		
Beck Depression Inventory		X				X	
Mini-Mental State Examination			X	X	X		
Reaction time tasks		X				X	X
Raven’s progressive matrices		X				X	
3-Back task		X				X	X
Mental rotation task		X				X	X
Digit span tasks		X				X	X
Five-point test						X	
Stop-switching task		X				X	X
Matchstick task						X	
Video gaming skills		X				X	

#### Neuropsychological Assessment

The participants were assessed using eight tasks (as listed in Table 2 and described in Table 3) to obtain a comprehensive measure of their cognitive abilities, with a special focus on executive functions and working memory. Not all tasks were administered at each assessment point due to the high risk of practice effects associated with some of them (e.g., the matchstick task; see Table 2). Except for the five-point test, all tasks were programmed using the E-Prime 2.0 software and computer-administered (for a full description see Palaus, 2018).

**Table 3:** Neuropsychological assessment tasks.

Task (in the order administered)	Description and assessed cognitive functions	Collected data
Reaction time tasks (Johnson et al., 1985)	Three short visual reaction time tasks (simple, direction choice and color choice), to assess processing speed	Accuracy and reaction times
Raven’s progressive matrices (Raven, 1936; Raven and Court, 2014)	Two parallel versions (for the Pre and Post1 assessments) of standard Raven’s progressive matrices, to measure general intelligence	Accuracy and reaction times
3-back task (based on Salat et al., 2002)	Visual continuous performance task, to measure working memory	Accuracy, reaction time, d’ sensitivity index [d’ = Z(hit rate) − Z(false alarm rate) as explained in Haatveit et al., 2010]
Mental rotation task (Shepard and Metzler, 1971)	Replicated Sheppard’s mental rotation task using 3D objects, to measure visuospatial skills	Accuracy and response time
Digit span task (Wechsler, 2008)	A computerized version of the WAIS-IV digit span test presented acoustically, to measure attention span and short-term memory (forward span) and working memory (backward span)	Digit span (forward and backward)
Stop-switching task (Obeso et al., 2013)	Speed task combining go, stop, and switch trials, to measure inhibition and task-switching components of executive functions	Accuracy and reaction time for go, stop and switch trials, and stop-signal reaction time (SSRT)
Five-point test (Tucha et al., 2012)	The paper-and-pencil task involving the connection of patterns consisting of 5 dots under a time constraint, to measure the ability to generate alternative solutions to a problem	Accuracy
Matchstick test (Knoblich et al., 1999)	Math problem-solving task involving Roman numerals made of sticks, as a measure of insight	Accuracy, reaction time

#### Video Game Training

For the video game training we used *Super Mario 64*, a video game created by Nintendo in 1996. *Super Mario 64* is a 3D platform game emphasizing exploration and puzzle-solving, requiring planning ability and goal-oriented behaviors. This video game was chosen because its use has shown a correlation with structural changes in the brain, i.e., an increase in cortical thickness of the right DLPFC, right hippocampal formation, and bilateral cerebellum, regions associated with executive functioning, spatial memory, and fine motor skills (Kühn et al., 2013).

Before and after the training, we assessed each participant’s video gaming skills by getting them to play an alternative version of the same game used in the training sessions for 15 min. We used the same level in both assessments. In particular, we assessed visuo-manual coordination, goal-oriented behavior, exploratory behavior, and behavior on facing obstacles and goal achievement (e.g., defeating an enemy, finding a hidden object, reaching a section of a level, etc.). This assessment was performed using a scale created *ad hoc*.

During training, participants played for 1.5 h at the rate of one session a day for 10 consecutive days excluding weekends (total 15 h) under the supervision of a researcher. Game sessions were also video recorded for later analysis. The only instructions given to participants were on how to use the controller and on what the main goals of the game were at the beginning of the training; otherwise, they played as they wished. The number of goals per session, the number of tries per goal, and the time needed to achieve each goal were measured. Overall performance was calculated by dividing the total number of goals achieved during the entire training period by the total number of attempts (each attempt ended either when the participant “lost-a-life” or achieved a goal).

The gaming experience was measured subjectively at each video game training session using a Likert scale of 1–5. Participants scored *motivation* (their desire to play) before playing, and *fun* (enjoyment during gameplay) and *frustration* (dissatisfaction during gameplay) after playing and before TMS.

#### Transcranial Magnetic Stimulation

A total of 10 TMS sessions were conducted, one after each video game training session. In each TMS session, brief pre- and post-stimulation assessments were made, consisting of the Mini-Mental State Examination (MMSE; Lobo et al., 2002), well-being, and acute substance consumption (only pre-stimulation) to detect and prevent any possible adverse effects of the stimulation. The TMS procedures complied with the international safety guidelines (Rossi et al., 2009), and none of the participants wore eye makeup to avoid local pain in the orbital area (Redolar-Ripoll et al., 2015).

**Figure 2:**
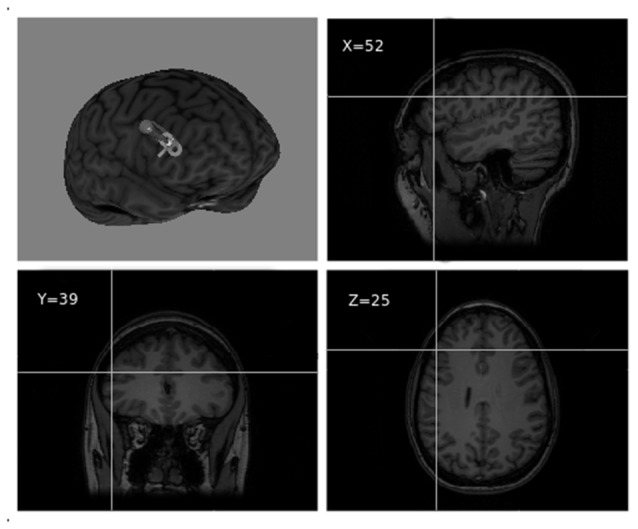
Right dorsolateral prefrontal cortex (DLPFC) TMS target visualized over a 3D MRI reconstruction of a participant’s head (reproduced with participant’s permission).

The stimulation target was set to Montreal Neurological Institute (MNI) coordinates *x* = 52, *y* = 39, *z* = 25, corresponding to the point of maximum grey matter increase in the right DLPFC as a result of training in the video game (Kühn et al., 2013; see Figure 2). Coordinates were individually adjusted for each participant based on their structural MRI. For target location and TMS guidance, we used the BrainSight 2 neuronavigation system (Rogue Research, Montreal, Canada).

Intermittent theta-burst stimulation (iTBS; Huang et al., 2005) was delivered following the international guidelines at 80% of the active motor threshold (Huang et al., 2005; Rossi et al., 2009; Suppa et al., 2016), using a Magstim Super Rapid 2 stimulator and a 70-mm figure-of-eight coil (Magstim Company Ltd., Whitland, UK). This stimulation protocol was chosen for its ability to induce long-term potentiation-like effects over the cerebral cortex (Goldsworthy et al., 2012) lasting up to 20 min (Suppa et al., 2016), with shorter stimulation times compared to other repetitive TMS protocols, but having similar effectiveness on clinical populations (e.g., Blumberger et al., 2018; Philip et al., 2019; Phillips et al., 2019). A total of 600 pulses were delivered in 50-Hz triplets repeated at 5 Hz (i.e., every 200 ms; Huang et al., 2011). This protocol involves repeating 2-s blocks of pulses interleaved with 8 s without stimulation 20 times during 200 s.

The sham stimulation group received the same stimulation protocol, except that the coil was tilted 90° over the vertex so that stimulation did not reach the cerebral cortex (Kaminski et al., 2011; Sandrini et al., 2011).

### Data Analysis

We analyzed age differences between groups using Student’s *t*-test (for comparisons of two groups) and one-way ANOVA (for comparisons of four subgroups). Gender differences between groups were calculated through Pearson’s chi-square test.

Fitness to parametric assumptions was checked for all variables. To assess the normality of data distribution, the Shapiro–Wilk test was used. The homogeneity of variances was contrasted through the Levene’s test of equality of variances. Lastly, to test sphericity, Mauchly’s test was used when there were three levels of a repeated measure.

When parametric assumptions were not satisfied, task scores were transformed, and/or alternative non-parametric tests were employed, as explained below.

To compare video gaming and cognitive performance between Active and Sham groups, and taking into account the assessment time points for the tasks (2 or 3 depending on the task), a repeated measures general linear model (rm GLM) was implemented for each variable. Significant effects and interactions were followed-up using paired *t*-tests Bonferroni-corrected for multiple comparisons. In measurements with just a single assessment point, the differences between groups were analyzed using the Student’s *t*-test. To study differences between groups for variables that were not normally distributed, task scores were transformed calculating new scores reflecting pre- vs. post-training changes. Two difference scores between time points were calculated as follows: (1) pre-assessment vs. a first post-assessment difference (Pre-Post1); and (2) pre-assessment vs. follow-up assessment difference (Pre-Post2). ANOVAs were used to compare those difference scores between groups when parametric assumptions were satisfied, otherwise, we used the Mann–Whitney’s U. When the distribution was normal, but equality of variances was not satisfied, we used Welch’s ANOVA to compare scores between groups.

Additionally, given the small sample size that might limit the study power, we performed a set of Bayesian analyses to determine whether a non-significant effect indicates a lack of intervention effect (Biel and Friedrich, 2018). In particular, we tested the relative plausibility of the alternative hypothesis (H1: synergistic effects of Active TMS+video game training on cognitive performance) over the null hypothesis (H0: the absence of such synergistic effects, i.e., equal performance of Active and Sham TMS groups after training). Thus, we calculated BF10 using the Bayesian counterpart’s tests of the analysis described above with a credible interval of 95%. We used the default Cauchy prior width of 0.707 provided by JASP (JASP Team, 2020) since we did not have previous data to establish an informed prior. The models used for the analysis were compared to the model containing the grand mean and the random factors, called the null model. In the case of the independent sample *t*-test (Student or Mann–Whitney, see Supplementary Table S2) we use unidirectional hypothesis tests expecting cognitive improvement in the Active compared to the Sham TMS group.

As explained in the “Design” section, after performing the main comparison between the Active and Sham groups, and based on previous literature on video games showing that personal variables account for a sizeable portion of the variance (see Palaus et al., 2017), additional analyses were performed to observe the possible relationship between individual variables and cognitive changes. Logistic regression accounting for age, gender, and the early video gaming experience were performed for each dependent variable. The results indicated that early video gaming experience and gender were potentially linked to cognitive performance results, with video gaming experience more directly related to changes in executive functions (see “Results” section).

Therefore, an rm GLM was used to compare the variables significantly influenced by early video game experience for the four subgroups resulting after combining stimulation type and early video gaming experience (Active+Exp, *n* = 6; Active+NoExp, *n* = 8; Sham+Exp, *n* = 6; and Sham+NoExp, *n* = 7). Parametric assumptions were also assessed for each variable for the four groups and we implemented the same procedures as above when assumptions were not met. But when, after transforming the scores, parametric assumptions were still not met, we used the Kruskal–Wallis H test to compare the four four subgroups, and when significant differences were found we used Mann–Whitney’s U to compare the groups in pairs. Since performance for one of the variables was also predicted by gender (besides gaming experience), gender was included as a covariate in the corresponding analysis.

Finally, to control for the potential influence of the subjective gaming experience on video game performance and potential stimulation effects (Maraver et al., 2016), the Student’s *t*-test was used to compare the Active and Sham groups for the motivation, fun, and frustration variables.

The dataset generated and analyzed during the current study, together with an explanatory readme file, is available in the institutional repository of the Universitat Oberta de Catalunya (O2), public URL: http://hdl.handle.net/10609/100246 (Palaus et al., 2019). All the frequentist analyses were performed using SPSS version 23 (IBM Software Group, IL, USA), and all the Bayesian analyses were performed in JASP computer software, version 0.12.2 (JASP Team, 2020).

## Results

### Demographic Data

Age was not significantly different between the Active and Sham groups (*t*_(25)_ = 0.35, *p* = 0.731) or between the four subgroups taking into account video gaming experience (*F*_(3,23)_ = 0.89; *p* = 0.457). There were no significant gender differences between the two initial groups (Active vs. Sham: $\chi_{\left(1\right)}^{2}$ = 0.40; *p* = 0.842) or the four subgroups (Active+Exp, Active+NoExp, Sham+Exp, Sham+NoExp, $\chi_{\left(1\right)}^{2}$ = 6.59; *p* = 0.086). Moreover, the number of participants with early video gaming experience was not significantly different between the Active and Sham groups ($\chi_{\left(1\right)}^{2}$ = 0.03; *p* = 0.863).

### Effects of the Transcranial Magnetic Stimulation

#### Frequentist Analyses

When comparing video game performance (after the 10 sessions of training and the video game skills before and after training; i.e., near transfer) and cognitive test performance (i.e., far transfer) for the Active (*n* = 14) and Sham (*n* = 13) groups, most differences were not statistically significant. See Table 4 for further information on tests and significance levels for each contrast and see Supplementary Table S1 for descriptive statistics of each variable (mean, standard deviation, and confidence interval). Here we will only report the significant results and their *post hoc* analyses.

**Table 4:** Statistical tests, statistics, and significance levels for comparisons of video games and cognitive tests performance for the Active vs. Sham groups.

					Main effects		
Test	Variable	Assessment time points	Scores	Statistical test	Group	Time	Interaction
Video game performance	10-session training	1	Direct	Mann–Whitney	*U* = 86.5, *p* = 0.827	-	-
	Pre vs. post skills	2	Direct	GLM	*F*_(1,25)_ = 0.121, *p* = 0.731	***F*_(1,25)_ = 101.95, *p* < 0.001**	*F*_(1,25)_ = 0.757, *p* = 0.393
RT	Simple	3	Direct	GLM	*F*_(1,25)_ = 0.04; *p* = 0.839	***F*_(2,50)_ = 6.33; *p* = 0.004**	***F*_(2,50)_ = 4.45; *p* = 0.017**
	Direction choice	3	Transformed (Post1-Pre, Post2-Pre)	ANOVA	-	-	*F*_(1,25)_ = 0.953, *p* = 0.338 *F*_(1,25)_ = 2.663, *p* = 0.115
	Color choice	3	Transformed (Post1-Pre, Post2-Pre)	ANOVA	-	-	*F*_(1,25)_ = 1.459, *p* = 0.238 *F*_(1,25)_ = 0.927, *p* = 0.345
Digits	Forward	3	Direct (Pre vs. Post1) Transformed (Post2-Pre)	GLM ANOVA	*F*_(1,25)_ = 0.015, *p* = 0.903	***F*_(1,25)_ = 6.156, *p* = 0.020** -	*F*_(1,25)_ = 0.008, *p* = 0.927 *F*_(1,25)_ = 0.037, *p* = 0.849
	Backward	3	Transformed (Post1-Pre, Post2-Pre)	ANOVA	-	-	*F*_(1,25)_ = 0.028, *p* = 0.867 *F*_(1,25)_ = 0.048, *p* = 0.828
3-back	Score	3	Transformed (Post1-Pre, Post2-Pre)	ANOVA	-	-	*F*_(1,25)_ = 0.851, *p* = 0.365 *F*_(1,25)_ = 0.1.085, *p* = 0.308
	RT	3	Direct (Greenhouse-Geisser correction)	GLM	*F*_(1,25)_ = 0.123, *p* = 0.729	***F*_(1.5,37.6)_ = 4.852 *p* = 0.021**	*F*_(1.50,37.61)_ = 4.852 *p* = 0.583
	d’	3	Direct	GLM	***F*_(1,25)_ = 5.526, *p* = 0.027**	*F*_(2,50)_ = 2.466, *p* = 0.095	*F*_(2,50)_ = 0.016, *p* = 0.984
Mental rotation	Score	3	Transformed (Post1-Pre, Post2-Pre)	ANOVA	-	-	*F*_(1,25)_ = 0.830, *p* = 0.371 *F*_(1,25)_ = 0.011, *p* = 0.917
	RT	3	Transformed (Post1-Pre, Post2-Pre)	Mann–Whitney	-	-	*U* = 54.00, *p* = 0.073 *U* = 69.00, *p* = 0.286
Stop-switching	Go score	3	Transformed (Post1-Pre, Post2-Pre)	Mann–Whitney (Post1-Pre) ANOVA (Post2-Pre)	- -	- -	*U* = 87.500, *p* = 0.864 *F*_(1,25)_ = 0.014, *p* = 0.907
	Go RT	3	Direct	GLM	*F*_(1,25)_ = 0.175, *p* = 0.679	***F*_(2,50)_ = 3.346, *p* = 0.043**	*F*_(2,50)_ = 0.603, *p* = 0.551
	Stop score	3	Transformed (Post1-Pre, Post2-Pre)	ANOVA	-	-	*F*_(1,25)_ = 1.134, *p* = 0.297 *F*_(1,25)_ = 1.326, *p* = 0.260
	Stop signal RT (SSRT)	3	Direct (Pre vs. Post1) Transformed (Post2-Pre)	GLM Mann–Whitney	*F*_(1,25)_ = 0.089, *p* = 0.767 -	***F*_(1,25)_ = 4.298, *p* = 0.049** -	*F*_(1,25)_ = 0.012, *p* = 0.913 *U* = 75.00, *p* = 0.438
	Switch score	3	Direct	GLM	*F*_(1,25)_ = 0.015, *p* = 0.902	***F*_(2,50)_ = 5.880, *p* = 0.005**	*F*_(2,50)_ = 0.023, *p* = 0.977
	Switch RT	3	Direct	GLM	*F*_(1,25)_ = 1.015, *p* = 0.321	***F*_(2,50)_ = 3.819, *p* = 0.029**	*F*_(2,50)_ = 0.213, *p* = 0.809
Raven	Score	2	Transformed (Post1-Pre)	Mann–Whitney	-	-	*U* = 81.500, *p* = 0.638
	RT	2	Direct	GLM	***F*_(1,25)_ = 7.412, *p* = 0.012**	*F*_(1,25)_ = 2.096, *p* = 0.160	*F*_(1,25)_ = 0.592, *p* = 0.449
Matchstick	Accuracy	1	Direct	*t*-test	*t*_(25)_ = 0.30, *p* = 0.763	-	-
	RT (correct answers)	1	Direct	*t*-test	*t*_(25)_ = 0.18, *p* = 0.861	-	-
Five-point		1	Direct	*t*-test	*t*_(25)_ = 0.94, *p* = 0.358	-	-

*Note: Statistically significant results are marked in bold. GLM, General linear model; RT, Reaction time*.

Some variables showed changes over time (main effect of time) that were similar for all groups, probably due to training and practice effects. Since our main interest was to compare the effects of TMS combined with video game training, that main effect of time was not specifically explored but was observed as part of the GLM analysis results and thus is only reported for variables that were normally distributed. In particular, we observed improvement over time in video game skills (*F*_(1,25)_ = 101.95, *p* = 0.000, $\eta_{\text{p}}^{2}$ = 0.80), digits forward (Pre vs. Post1 assessments *F*_(1,25)_ = 6.16, *p* = 0.020, $\eta_{\text{p}}^{2}$ = 0.20), 3-back task reaction times (*F*_(1.5,37.61)_ = 4.85 *p* = 0.021, $\eta_{\text{p}}^{2}$ = 0.16), and accuracy (*F*_(2,50)_ = 5.88, *p* = 0.005, $\eta_{\text{p}}^{2}$ = 0.19). In the Stop-switching task, Switch trials reaction times decreased (*F*_(2,50)_ = 3.82, *p* = 0.029, $\eta_{\text{p}}^{2}$ = 0.13), but Go reaction times (*F*_(2,50)_ = 3.35, *p* = 0.043, $\eta_{\text{p}}^{2}$ = 0.12) and stop signal reaction times (SSRT; Pre vs. Post1 *F*_(1,25)_ = 4.3, *p* = 0.049, $\eta_{\text{p}}^{2}$ = 0.15) increased. In the case of the simple reaction time task, results also deteriorated over time (*F*_(2,50)_ = 6.33; *p* = 0.004, $\eta_{\text{p}}^{2}$ = 0.20). It should be noted, however, that this was also the only task showing significant interaction effects between the stimulation group and the assessment time points (*F*_(2,50)_ = 4.45; *p* = 0.017, $\eta_{\text{p}}^{2}$ = 0.15; see Table 4). *Post hoc* analyses revealed that participants in the Active group had slower reaction times in the Post2 assessment compared to both the Pre (*p* = 0.005, mean difference = 28.3, 95% CI 7.5–49.1) and the Post1 assessment (*p* = 0.001, mean difference = 25.6, 95% CI 10.2–41.1). Interestingly enough, this effect was not observed for the more complex reaction time tasks (color and direction reaction time: *p* > 0.115; see Table 4).

We also found statistically significant differences between the Active and Sham groups in some tasks (main effect of the group), given the absence of interaction effect between group and time, this was probably due to differences in baseline performance. This effect was observed for the 3-back d’ score (*F*_(1,25)_ = 5.53, *p* = 0.027, $\eta_{\text{p}}^{2}$ = 0.18) and for the time taken to complete Raven’s progressive matrices (*F*_(1,25)_ = 7.41, *p* = 0.012, $\eta_{\text{p}}^{2}$ = 0.23). In both cases, the Active group showed better scores than the Sham group and significant differences between groups at baseline (*ps* < 0.043).

#### Bayesian Analyses

The comparisons between the Active and Sham TMS stimulation groups using Bayesian methods yield very similar results than the ones obtained when performing the same comparisons through frequentist statistical analyses. However, the main effect of time only showed strong evidence in favor of the alternative hypothesis (i.e., BF greater than 10 following Jeffreys, 1961) for the improvement in video game skills (BF_10_ = 6.575 × 10^7^, error% = 2.163). The rest of the variables showing changes in the previous analyses obtained weak (BF 1–3) to moderate (BF 3–10) evidence (see Supplementary Table S2 in the Supplementary Material). The only significant group by time interaction effect found through frequentist analysis for the simple reaction time task could not be replicated here (BF_incl_ = 0.359; see Supplementary Table S2).

Regarding the few previously observed differences between groups, the evidence in favor of the alternative hypothesis was also weak to moderate. Overall, the Bayesian analysis reinforces the idea that the non-significant effects found *via* frequentist analyses support the null hypothesis.

### Logistic Regression of Individual Variables

Age, gender, and early video gaming experience were explored through logistic regression analysis (using enter method), to find out whether these variables could predict the changes in cognitive performance—most especially in executive functions and working memory—after training (i.e., predictors over the difference scores between pre- and post-training assessments).

The model was statistically significant for the 3-back task accuracy (*F*_(3,23)_ = 3.36, *p* = 0.036, adjusted R^2^ = 0.21) and the d’ index (*F*_(2,23)_ = 5.23, *p* = 0.007, adjusted R^2^ = 0.33); and the Stop-switching task SSRT (*F*_(2,23)_ = 5.55, *p* = 0.005, adjusted R^2^ = 0.34). In particular, changes observed in the d’ index for the 3-back task were predicted only by early video gaming experience (beta = 0.66, *t*_(26)_ = 3.51, *p* = 0.002), but not by age or gender (*p*s > 0.788). Similar results were obtained for the accuracy score for the 3-back task were only the *p*-value was close-to-significant for early video gaming experience (beta = 0.41, *t*_(26)_ = 2.01, *p* = 0.056). SSRT changes were influenced by both early video gaming experience (beta = −0.70, *t*_(26)_ = −3.78, *p* = 0.001) and gender (beta = −0.57, *t*_(26)_ = 3.15, *p* = 0.004).

### Effects of Video Gaming Experience

To further explore the influence of early video gaming experience and gender on cognitive performance, we first determined whether there was a gender imbalance between experienced (*n* = 12) and non-experienced (*n* = 15) participants, finding that the difference was statistically significant ($\chi_{\left(1\right)}^{2}$ = 6.24; *p* = 0.013), with more male participants having early video gaming experience (69.23%) than women (21.43%).

Then we explored the influence of video gaming experience on the d’ score for the 3-back task and the SSRT for the Stop-switching task by dividing the Active and Sham groups into two subgroups each of experienced and non-experienced participants, resulting in the four subgroups described above (see the “Design” and “Demographic Data” sections). For the SSRT we also included gender as a covariate based on the results of the logistic regression.

**Figure 3:**
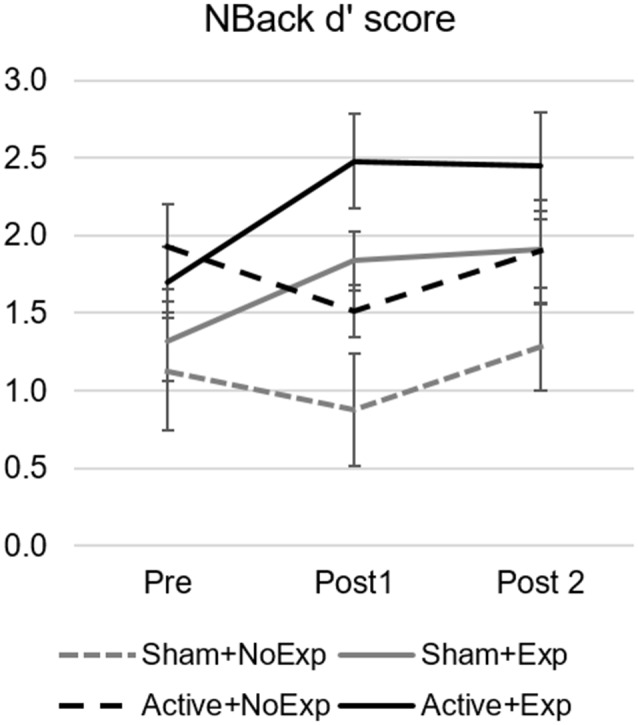
3-back d’ score for the four subgroups and the three assessment points. Error bars indicate the standard error of the mean. The change between Pre and Post1 assessments was statistically significant for the Active+Exp group (solid black line) compared to the two groups without video gaming experience (dashed lines). Differences between the Pre and Post1 assessment were also significant for the two groups with early video gaming experience (solid lines).

For the 3-back task, results showed that, when comparing the change in d’ scores between pre and post1 assessment among groups, there was a statistically significant difference (*F*_(3,23)_ = 5.6, *p* = 0.005, $\eta_{\text{p}}^{2}$ = 0.42), where performance in the Active+Exp group was superior to that of Sham+NoExp, despite starting from an equal baseline (see Figure 3). *Post hoc* analysis revealed differences between the Active+Exp subgroup and the two subgroups without early video gaming experience, namely, Sham+NoExp (*p* = 0.048) and Active+NoExp (*p* = 0.012). Furthermore, *t*-tests comparing Pre vs. Post1 3-back d’ scores within group, revealed significant differences for the two subgroups with early video gaming experience (Sham+Exp: *t*_(5)_ = −3.28, *p* = 0.022 and Active+Exp: *t*_(5)_ = −3.36, *p* = 0.020), but not for the two subgroups without early video gaming experience (Sham+NoExp: *t*_(6)_ = −1.32, *p* = 0.236 and Active+NoExp: *t*_(7)_ = 1.33, *p* = 0.225).

**Figure 4:**
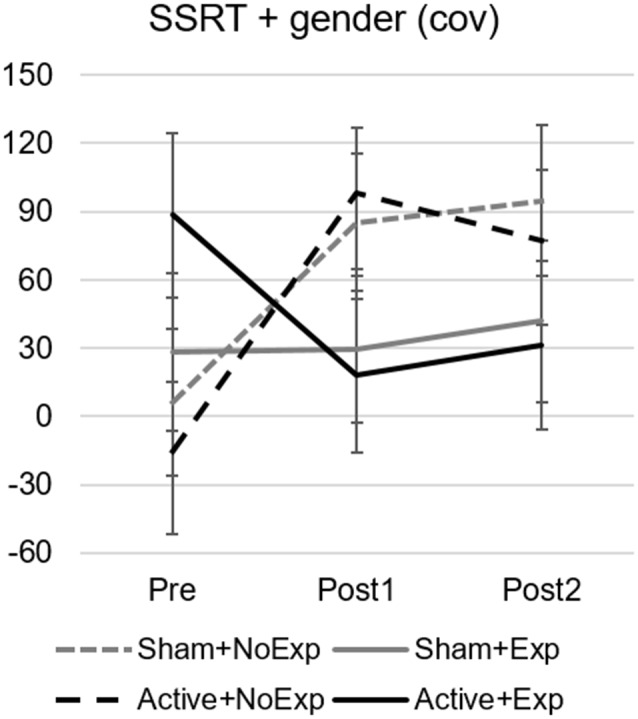
Stop signal reaction times (SSRT) for the Stop-switching task with gender as a covariate for the four subgroups and the three assessment points. Error bars indicate the standard error of the mean. Differences were significant between the Pre and Post1 assessments in the two groups without early video gaming experience (dashed lines).

For the SSRT, the GLM revealed a main effect of time (*F*_(2,44)_ = 7.71, *p* = 0.001, $\eta_{\text{p}}^{2}$ = 0.26), an interaction effect of time by gender (*F*_(2,44)_ = 5.37, *p* = 0.008, $\eta_{\text{p}}^{2}$ = 0.20), and a close-to-significant interaction between time and group (*F*_(6,44)_ = 2.2, *p* = 0.061, $\eta_{\text{p}}^{2}$ = 0.23; see Figure 4). This last interaction effect was due to significant differences between Pre and Post1 assessments for the two subgroups without video gaming experience (Sham+NoExp, *p* = 0.033 and Active+NoExp, *p* = 0.001).

### Subjective Gaming Experience

The levels of motivation before and fun and frustration after the training were not significantly different between the Active and Sham groups (*p*s > 0.483) or between the four subgroups (*p*s > 0.522), not even for gender (*p* = 0.264).

## Discussion

This research aimed to assess whether a dual intervention based on video game training and non-invasive brain stimulation could enhance cognitive function in terms of: (1) specific cognitive abilities directly trained during the intervention (i.e., video gaming skills, near-transfer effect); and (2) other cognitive functions not directly trained (i.e., generalization or far-transfer effect). Results showed very limited changes that were mainly related to the near-transfer effect, i.e., variables related to video gaming skills.

The near-transfer effect assessed using an alternative version of the video game that participants played before and after the intervention period, was present in both the Active and Sham stimulation groups. This result combined with the fact that there was no difference between those groups in video game achievements at the end of the training period, seems to indicate that the near-transfer effect was only due to the video game training and not mediated by TMS. To our knowledge, there is no literature in which TMS has shown improvements in video gaming performance, although some recent studies have documented positive near-transfer results using transcranial direct current stimulation (e.g., Hsu et al., 2015; Looi et al., 2016). Moreover, the literature showing cognitive improvement in healthy participants using TMS is limited and some studies have shown negative results or subtle effects (e.g., Gaudeau-Bosma et al., 2013; Viejo-Sobera et al., 2017). Even more, the number of studies showing a lack of effect of TMS on cognition may be hard to determine due to strong publication bias.

Regarding the effect of the training period on the evaluated cognitive domains (far-transfer), we observed an improvement in certain cognitive control tasks: digits forward, 3-back task, and accuracy and reaction times for switching trials in the stop-switching task. In contrast, we observed a deterioration in reaction times in go trials and the SSRT of this task. All these effects were present in both the Active and Sham groups, probably due to the video game training and the practice effects present in the neuropsychological tasks, observing, again, the lack of effect of the TMS stimulation. In any case, the near-transfer effects seemed more meaningful since the observed effect size (partial eta squared) in this case was much bigger than that observed in the possible far-transfer effects (0.80 vs. 0.12–0.23, respectively), and the Bayesian analyses only showed weak to moderate evidence to support the alternative hypothesis of far-transfer effect.

Since we did not find an effect of TMS on cognitive performance, we explored other variables that could influence the results. This analysis led us to observe that early video gaming experience may have mediated the improvement in certain cognitive functions. In particular, participants with early video gaming experience improved working memory performance (as evidenced by the increase in the d’ index of the 3-back task). This was a surprising result since far-transfer effects on visual working memory tasks as a result of video gaming play have not been widely documented in the literature. We have found one study in which working memory performance was enhanced after video game training in a first-person shooter action game (compared to a simulation-strategy game; Blacker et al., 2014), but another one, which trained in a real-time strategy game failed to find such an effect (Basak et al., 2011). Regarding inhibitory control, we observed that participants without early video gaming experience increased their reaction times while accuracy was maintained in the SSRT of the Stop-Switching task. This is most likely due to a change of strategy to avoid the interference between go, stop, and switch trials, making participants more cautious towards their responses. Experienced players, however, did not show this change in response strategy, maintaining lower reaction times after the training period. This lack of improvement and maintenance of inhibitory ability in experienced gamers has been shown in previous studies (Whitlock et al., 2012; Colzato et al., 2013; Steenbergen et al., 2015).

The similar near- and far-transfer effects found in both stimulation groups, together with the effects observed only in participants with early video gaming experience, corroborate the observation that playing video games may have an impact on cognitive functions in the long term (41–43). The 10-day training period would have made it easier for experienced players to improve their performance in specific executive function domains. In any case, given the small sample size of the subgroups included in the comparison of experienced vs. not experienced gamers, these results should be interpreted as exploratory.

The observed lack of effect of TMS in any of the studied domains has several possible explanations. In the following paragraphs, we will explore different arguments and considerations previously documented in the literature to understand our results.

First, we must consider whether the stimulation protocol (iTBS after training) and the targeted brain region (the DLPFC) were the most appropriate for the specific goals of this research. Although iTBS protocol is still not widely used in research focused on cognitive enhancement, it was chosen for its ability to induce longer-lasting effects over the cerebral cortex with shorter stimulation times compared to rTMS (Goldsworthy et al., 2012; Suppa et al., 2016). A recent systematic review and meta-analysis that assessed the reliability and effectiveness of theta-burst stimulation protocols applied to the prefrontal cortex in healthy participants (Lowe et al., 2018), has shown the variability in the effects. iTBS can somehow modulate executive control, but its effectiveness seems to be task-dependent, being greater for working memory paradigms. Nevertheless, only eight studies have used this paradigm to improve executive functions, so the results should be interpreted with caution. Regarding the time of stimulation, we decided to apply it after the training session to improve the effects of the training as shown in animal models (Redolar-Ripoll et al., 2002). Nevertheless, stimulation before performing a specific task is also a common practice in human studies and this shift in time could have affected the results.

We should also consider that the effect of TMS in healthy samples is potentially explained by inter- and intra-individual variables (Hinder et al., 2014; López-Alonso et al., 2014; Suppa et al., 2016; Jannati et al., 2017). These variables include genetic factors (e.g., Cheeran et al., 2008; Li Voti et al., 2011; Mori et al., 2011; Lee et al., 2014), cortical networks organization (Nettekoven et al., 2014, 2015) and age (Müller-Dahlhaus et al., 2008), among other factors. Moreover, the number of sessions can also be a limiting factor in achieving the desired results. Although effects have been observed after 10 sessions of stimulation, interventions in clinical populations usually require more sessions to observe more consistent long-term effects. For example, the mean number of sessions for treating depression is 17, with a great degree of variability among studies, typically ranging from 9 to 25 sessions (Berlim et al., 2017).

Regarding the target for the stimulation, the right DLPFC, is a relatively large area; nonetheless, the figure-of-eight coil and the navigated stimulation increase the focus and precision of the target location. Given the extensive connectivity of the DLPFC (Sepulcre et al., 2012) and the involvement of different regions of this area in the measured functions, possibly the highly specific target was not directly responsible for the neural processes underlying the cognitive abilities assessed by our tasks. We selected our target based on structural changes observed after playing this particular video game (Kühn et al., 2014) and its involvement in executive functions and working memory, but perhaps our stimulation protocol and/or target did not affect the functional circuits involved in cognitive changes after video game practice (Strenziok et al., 2014; Richlan et al., 2018). This would have been particularly relevant since TMS might be more likely to induce functional than structural changes, especially after a short intervention period.

Brain state-dependency merits special interest, since “any induced neural activity occurs in the context of a baseline neural activity” (Silvanto and Pascual-Leone, 2008). It has been shown that the effects of TMS could be qualitatively modulated by the manipulation of the brain state before the stimulation (Silvanto et al., 2017; e.g., Silvanto and Cattaneo, 2017). In our study, we administered stimulation together with cognitive training to take advantage of the state-dependency phenomenon, and all participants received stimulation immediately after finishing the video gaming period. However, we did not implement any specific control of the ongoing mental state of participants immediately before or during stimulation. Thus, the neural activity could certainly have differed in the participants, leading to uncontrolled or null effects of the stimulation.

Moreover, and considering our results, we must also take into account that the brain network organization of experienced vs. non-experienced gamers has probably led to different brain states while gaming and, thus, when receiving the stimulation after gaming. This might have constituted an advantage for the experienced gamers in terms of responding positively to the stimulation, explaining why the experienced participants who received active TMS showed a greater improvement in the 3-back task compared to both experienced and non-experienced sham groups, and with the active TMS non-experienced group.

Lastly, we need to reflect on the ceiling effect of cognitive enhancement in healthy subjects. In neuropsychology, patients with neurological diseases or psychiatric patients usually have some room for improvement, but in healthy individuals who already perform at, or close to, their full potential, it is difficult to achieve cognitive enhancement. The effects of TMS are linked to the baseline performance of participants, with lower baseline scores associated with higher cognitive facilitation (Silvanto et al., 2018). In the same meta-analysis mentioned above (Lowe et al., 2018), the authors point out that the largest effect size was observed for an older adult population, supporting the idea that iTBS may be more effective in addressing cognitive decline or impairment in clinical or vulnerable populations than in enhancing cognition in healthy ones. Along the same lines, Looi et al. (2016) reported better results for subjects who performed worse at baseline. In our study, participants were healthy, young, and with high education levels, which would leave little room for cognitive improvement. Overall, these and previous results underscore the importance of reconsidering whether efforts in non-invasive brain stimulation research should be aimed at enhancing cognitive performance in healthy individuals, and rise some ethical concerns given the possibly greater potential of these techniques for clinical populations. Nevertheless, we should not forget that the ultimate goal of this study, and many others involving healthy participants, is to apply the knowledge gained in these samples to clinical ones, rather than trying to benefit healthy individuals in no need for these technologies. In any case, the ethical debate must be always present in neuroscience research and especially in the non-invasive stimulation field.

## Conclusions

This study aimed to test the synergistic effect of the combination of non-invasive brain stimulation and video game training in cognitive enhancement. However, our results did not support this hypothesis, showing an absence of combined effects on cognitive performance. Due to this observed lack of effect, we explored other variables that could have influenced our results showing that early video gaming experience had an impact on the improvement of certain cognitive functions. This result supports the idea that video gaming may modulate cognitive functions in the long term.

Contrary to most studies in the literature where iTBS led to improvements in cognitive tasks at the end of the stimulation, we did not assess cognitive performance immediately after stimulation, as our goal was to determine the presence of transfer effects of the training period. In this scenario, the application of iTBS to the right DLPFC seems to be ineffective in achieving long-term cognitive improvements in healthy patients. Possible explanations may be the poor reliability and effectiveness of the iTBS protocol, highly localized stimulation in a large and widely connected brain area, inter- and intra-individual variability, brain state dependency phenomenon, the ceiling effect of cognitive enhancement in healthy subjects, or a combination of some or all of these factors.

Despite not achieving the desired effects of the stimulation, our results, although exploratory, provide valuable information regarding the limitations of stimulating healthy brains and the possible beneficial effects of exposure to video games.

## Data Availability Statement

The dataset generated and analyzed during the current study is available in the institutional repository of the Universitat Oberta de Catalunya (O2), public URL: http://hdl.handle.net/10609/100246 (Palaus et al., 2019).

## Ethics Statement

The studies involving human participants were reviewed and approved by Universitat Oberta de Catalunya (UOC) Ethics Committee. The patients/participants provided their written informed consent to participate in this study.

## Author Contributions

MP, DR-R, and EM conceptualized and designed the study. MP designed the experimental tasks. MP and RV-S acquired and analyzed the data, and all authors interpreted the results and drafted and reviewed the manuscript. All authors have approved the submitted version and have agreed to both to be personally accountable for the author’s contributions and to ensure that questions related to the accuracy or integrity of any part of the work, even ones in which the author was not personally involved, are appropriately investigated, resolved, and the resolution documented in the literature.

## Conflict of Interest

The authors declare that the research was conducted in the absence of any commercial or financial relationships that could be construed as a potential conflict of interest.

## Abbreviations

BF: Bayes Factor
DLPFC: dorsolateral prefrontal cortex
Exp: participants with early video game experience
GLM: general linear model
iTBS: intermittent theta-burst stimulation
MRI: magnetic resonance imaging
NoExp: participants without early video game experience
rm GLM: repeated measures GLM
SSRT: stop-signal reaction time
TMS: transcranial magnetic stimulation
TMS+VG: TMS together with video game training.
