# Supplementary material for: Cognitive Enhancement via Neuromodulation and Video Games: Synergistic Effects?
Source: Front Hum Neurosci. 2020 Jun 19;14:235. doi: 10.3389/fnhum.2020.00235 (PMC7319101; doi:10.3389/fnhum.2020.00235)
Supplement: Supplementary file 1 [file Table_1.pdf]

Table S1. Data summary for each test on each of the 3 different assessment time points.

Test	Variable	Group	Pre		Post1		Post2	
			Mean ± SD	CI 95%	Mean ± SD	CI 95%	Mean ± SD	CI 95%
Video game performance	10-session training	Active	31.79 ± 27.72	15.78 - 47.79	-	-	-	-
		Sham	29.92 ± 24.63	15.04 - 44.81	-	-	-	-
	Pre vs. post skills	Active	8.57 ± 2.441	7.16 - 9.98	13.14 ± 3.483	11.13 - 15.15	-	-
		Sham	9.31 ± 2.810	7.61 - 11.01	13.15 ± 3.255	11.86 - 14.44	-	-
RT	Simple	Active	262.65 ± 32.74	243.75 - 281.55	265.32 ± 37.47	243.68 - 286.95	290.95 ± 43.18	266.02 - 315.88
		Sham	272.76 ± 24.66	257.85 - 287.66	270.91 ± 32.77	257.95 - 283.87	276.53 ± 34.44	255.71 - 297.34
	Direction choice	Active	320.34 ± 37.29	298.81 - 341.88	324.79 ± 38.45	302.59 - 346.99	339.78 ± 46.85	312.73 - 366.83
		Sham	347.40 ± 47.46	318.72 - 376.07	341.40 ± 45.38	341.40 - 45.38	340.11 ± 43.65	313.73 - 366.48
	Color choice	Active	455.70 ± 75.49	412.11 - 499.29	466.87 ± 79.98	420.70 - 513.05	475.56 ± 87.87	424.82 - 526.29
		Sham	468.03 ± 63.53	429.64 - 506.42	449.57 ± 37.13	427.13 - 472.01	462.88 ± 56.43	428.77 - 496.98
Digits	Forward	Active	9.43 ± 1.50	8.56 - 10.30	10.29 ± 2.43	8.88 - 11.69	10.57 ± 1.40	9.04 - 12.19
		Sham	9.31 ± 2.18	7.99 - 10.62	10.23 ± 2.09	8.97 - 11.49	10.62 ± 2.60	9.76 - 11.38
	Backward	Active	8.79 ± 2.72	7.21 - 10.36	9.07 ± 1.86	8.00 - 10.14	10.36 ± 2.87	8.70 - 12.02
		Sham	8.00 ± 2.52	6.48 - 9.52	8.38 ± 1.89	7.24 - 9.53	9.38 ± 2.96	7.60 - 11.17
3-back	Score	Active	50.00 ± 7.190	45.85 - 54.15	48.64 ± 12.822	41.24 - 56.05	51.57 ± 7.460	47.26 - 55.88
		Sham	48.15 ± 4.562	45.40 - 50.91	49.69 ± 6.250	45.92 - 53.47	51.46 ± 3.755	49.19 - 53.73
	RT	Active	873.47 ± 304.79	697.49 - 1049.45	903.50 ± 276.82	743.67 - 1063.33	813.32 ± 225.11	683.35 - 943.29
		Sham	943.85 ± 309.28	756.96 - 1130.74	918.80 ± 294.40	740.90 - 1096.70	833.99 ± 260.80	676.39 - 991.59
	d'	Active	1.83 ± 0.68	1.44 - 2.22	1.93 ± 0.76	1.49 - 2.36	2.13 ± 0.91	1.61 - 2.66
		Sham	1.21 ± 0.82	0.72 - 1.71	1.32 ± 0.89	0.78 - 1.86	1.86 ± 0.86	1.52 - 2.20
Mental rotation	Score	Active	.883 ± .086	.833 - .932	.911 ± .108	.849 - .973	.902 ± .101	.844 - .961
		Sham	.929 ± .048	.899 - .958	.942 ± .049	.913 - .971	.947 ± .041	.922 - .971
	RT	Active	7644 ± 3716	5498 - 9790	6250 ± 2478	4819 - 7680	4803 ± 2436	3396 - 6210
		Sham	8366 ± 3139	6469 - 10263	6186 ± 2325	4781 - 7591	4553 ± 1893	3804 - 5302

Test	Variable	Group	Pre		Post1		Post2	
			Mean ± SD	CI 95%	Mean ± SD	CI 95%	Mean ± SD	CI 95%
Stop-switching	Go score	Active	285.93 ± 2.018	284.76 - 287.09	286.50 ± 2.066	285.31 - 287.69	286.71 ± 1.437	285.88 - 287.54
		Sham	286.08 ± 1.706	286.08 - 1.706	286.54 ± 2.470	285.05 - 288.03	286.77 ± 1.166	286.06 - 287.47
	Go RT	Active	733.79 ± 144.97	650.08 - 817.49	768.67 ± 177.31	666.30 - 871.05	798.31 ± 176.25	696.55 - 900.07
		Sham	734.01 ± 129.03	656.04 - 811.99	734.67 ± 144.54	647.32 - 822.01	762.70 ± 140.16	678.00 - 847.40
	Stop score	Active	61.07 ± 7.07	56.99 - 65.15	64.00 ± 7.21	59.84 - 68.16	65.50 ± 5.96	62.06 - 68.94
		Sham	63.00 ± 7.45	58.50 - 67.50	63.92 ± 8.32	58.89 - 68.95	64.46 ± 7.89	59.69 - 69.23
	Stop signal RT (SSRT)	Active	27.79 ± 95.37	-27.28 - 82.85	64.58 ± 81.31	17.63 - 111.52	58.62 ± 86.80	8.50 - 108.74
		Sham	17.89 ± 79.27	-30.01 - 65.79	58.80 ± 76.34	12.67 - 104.93	69.34 ± 81.34	20.19 - 118.50
	Switch score	Active	53.71 ± 9.587	48.18 - 59.25	56.93 ± 10.637	50.79 - 63.07	58.57 ± 9.221	53.25 - 63.90
		Sham	54.46 ± 8.452	49.35 - 59.57	57.23 ± 10.248	51.04 - 63.42	58.77 ± 9.311	53.14 - 64.40
	Switch RT	Active	130.83 ± 76.96	86.39 - 175.26	109.67 ± 98.35	52.88 - 166.46	100.80 ± 119.67	31.70 - 169.90
		Sham	158.61 ± 66.88	118.20 - 199.02	148.00 ± 63.80	109.44 - 186.55	124.46 ± 58.28	89.24 - 159.68
Raven	Score	Active	27.00 ± 4.26	24.54 - 29.46	26.79 ± 3.19	24.94 - 28.63	-	-
		Sham	28.38 ± 1.71	27.35 - 29.42	27.62 ± 2.02	26.39 - 28.84	-	-
	RT	Active	15022 ± 5796	11675 - 18368	14165 ± 4907	11332 - 16998	-	-
		Sham	21184 ± 8045	16322 - 26046	18460 ± 4215	15913 - 21007	-	-
Matchstick	Accuracy	Active	5.00 ± 2.038	3.82 - 6.18	-	-	-	-
		Sham	5.23 ± 1.878	4.10 - 6.37	-	-	-	-
	RT (correct answers)	Active	47647 ± 17969	37272 - 58022	-	-	-	-
		Sham	48986 ± 21271	36132 - 61840	-	-	-	-
Five-point		Active	30.00 ± 10.054	24.20 - 35.80	-	-	-	-
		Sham	33.23 ± 7.596	28.64 - 37.82	-	-	-	-

Note: RT: Reaction time; SD: Standard deviation; CI: Confidence interval.
