# Supplementary material for: Cognitive Enhancement via Neuromodulation and Video Games: Synergistic Effects?
Source: Front Hum Neurosci. 2020 Jun 19;14:235. doi: 10.3389/fnhum.2020.00235 (PMC7319101; doi:10.3389/fnhum.2020.00235)
Supplement: Supplementary file 2 [file Table_2.pdf]

Table S2. Bayesian analysis for comparisons of video games and cognitive tests performance for the Active vs. Sham groups.

Test	Variable	Assessment time points	Scores	Bayesian Statistical test	Main effects (BF_{10})		Interaction
					Group	Time	
Video game performance	10-session training	1	Direct	Mann-Whitney	BF_{-0}=0.450, W=93.500, R^2=1.000	-	-
	Pre vs. post skills	2	Direct	rmANOVA	0.362, err%=1.607	**6.575×10^7^, err%=2.163**	BF_{incl} =0.478
RT	Simple	3	Direct	rmANOVA	0.470, err%=1.747	**6.488, err%=0.740**	BF_{incl} =0.359
	Direction choice	3	Transformed (Post1-Pre, Post2-Pre)	ANOVA	-	-	BF_{10}=0.510, err%=8.382×10^{-5}<br>BF_{10}=0.941, err%=0.003
	Color choice	3	Transformed (Post1-Pre, Post2-Pre)	ANOVA	-	-	BF_{10}=0.631, err%=0.003<br>BF_{10}=0.505, err%=4.316×10^{-5}
Digits	Forward	3	Direct (Pre vs. Post1)<br>Transformed (Post2-Pre)	rmANOVA<br>ANOVA	0.421, err%=0.679<br>-	**2.923, err%=0.877**<br>**-**	BF_{incl} =0.336<br>BF_{10}=0.363, err%=8.871×10^{-6}
	Backward	3	Transformed (Post1-Pre, Post2-Pre)	ANOVA	-	-	BF_{10}=0.362, err%=7.087×10^{-6}<br>BF_{10}=0.365, err%=1.188×10^{-5}
3-back	Score	3	Transformed (Post1-Pre, Post2-Pre)	ANOVA	-	-	BF_{10}=0.491, err%=2.558×10^{-5}<br>BF_{10}=0.535, err%=4.655×10^{-4}
	RT	3	Direct	rmANOVA	0.578, err%=2.085	**4.091, err%=0.595**	BF_{incl} =0.477
	d'	3	Direct	rmANOVA	**2.580, err%=1.243**	0.754, err%=1.247	BF_{incl} =0.351
Mental rotation	Score	3	Transformed (Post1-Pre, Post2-Pre)	ANOVA	-	-	BF_{10}=0.487, err%=4.792×10^{-5}<br>BF_{10}=0.360, err%=4.885×10^{-6}
	RT	3	Transformed (Post1-Pre, Post2-Pre)	Mann-Whitney	-	-	BF_{-0}=0.166, W=128.000, R^2=1.001

Test	Variable	Assessment time points	Scores	Bayesian Statistical test	Main effects (BF_{10})		Interaction
					Group	Time	
							BF_{-0}=0.247, W=113.000, R^2=1.001
Stop-switching	Go score	3	Transformed (Post1-Pre, Post2-Pre)	Mann-Whitney (Post1-Pre)<br>rmANOVA (Post2-Pre)	-<br>0.323, err%=1.979	-<br>0.361, err%=0.885	BF_{-0}=0.379, W=87.500, R^2=1.001<br>BF_{incl} =0.372
	Go RT	3	Direct	rmANOVA	0.563, err%=2.064	**1.387, err%=0.930**	BF_{incl} =0.466
	Stop score	3	Transformed (Post1-Pre, Post2-Pre)	ANOVA	-	-	BF_{10}=0.544, err%=6.710×10^{-4}<br>BF_{10}=0.548, err%=0.002
	Stop signal RT (SSRT)	3	Direct (Pre vs. Post1)<br>Transformed (Post2-Pre)	rmANOVA<br>Mann-Whitney	0.367, err%=1.445<br>-	**1.828, err%=4.149**<br>-	BF_{incl} =0.339<br>BF_{-0}=0.488, W=75.500, R^2=1.001
	Switch score	3	Direct	rmANOVA	0.480, err%=1.831	**9.174, err%=0.671**	BF_{incl} =0.376
	Switch RT	3	Direct	rmANOVA	0.673, err%=0.829	**2.037, err%=1.855**	BF_{incl} =0.361
Raven	Score	2	Transformed (Post1-Pre)	Mann-Whitney	-	-	BF_{-0}=0.520, W=100.500, R^2=1.000
	RT	2	Direct	rmANOVA	**4.538, err%=0.768**	0.639, err%=1.544	BF_{incl}=0.429
Matchstick	Accuracy	1	Direct	t-test	0.371, err%=3.012×10^{-5}	-	-
	RT (correct answers)	1	Direct	t-test	0.362, err%=7.664×10^{-6}	-	-
Five-point		1	Direct	t-test	0.496, err%=6.673×10^{-6}	-	-

Note: Bayesian probabilities higher than the null model are marked in bold. Credibility interval: 95.0%. BF: Bayes Factor; BF_{10}: evidence for the alternative hypothesis relative to the null hypothesis; BF_{-0}: evidence for the one-sided alternative hypothesis that group one < group two, relative to the null hypothesis, BF_{incl}: change from prior to posterior inclusion odds; err%: error percentage gives a numerical robustness of the result (the lower the err%, the greater numerical stability of the result); RT: Reaction time.
